# Challenges and constraints in the sustainability of poultry farming in Japan

**DOI:** 10.5713/ab.24.0675

**Published:** 2025-02-25

**Authors:** Haruhiko Ohtsu

**Affiliations:** 1 Institute of Livestock and Grassland Science, NARO, Tsukuba, Japan

**Keywords:** Broiler, Heat Stress, Japan, Layer, Poultry Farming

## Abstract

Poultry products such as chicken meat and eggs are among the most common and popular animal products in Japan. Recently, many chickens, such as broilers and layers, have been raised and their related product consumption has increased. However, the number of farms decreased, which is one of the major challenges faced by the Japanese poultry industry. Similar to that in other countries, high-pathogenicity avian influenza (HPAI) outbreaks negatively affected the distribution of poultry products. Low feed self-sufficiency in Japan is also a serious problem because the prices of diets and products are affected by the situation in foreign countries. Rice is a domestic ingredient of the poultry diet in Japan, and recently, its utilization has increased; however, concerns remain. Global warming likely affects the poultry industry in Japan negatively. The objective of this review is to illustrate the recent situation of the Japanese poultry industry, including 1) an overview; 2) the situation of influence of HPAI; 3) situation of ingredients for poultry diet; 4) utilization of rice in poultry diet; 5) heat stress in poultry. Overall, investigation of the effects of heat stress on physiology, such as the biological defense system, and its prevention, should be continued to prevent future decreases in productivity in the Japanese poultry industry.

## INTRODUCTION

Chicken meat and eggs are among the most common and popular poultry products globally. Broiler meat (without skin) has a lower amount of fat than that of beef and pork [1]. Chicken eggs contain well-balanced amino acids and are a good source of energy, vitamins, and minerals [2]. Therefore, poultry products are consumed frequently, and farmed globally, including in Japan [3].

The poultry industry in Japan grew after World War II, with a drastic increase in the number of farms [4]. The number of farms with layer chickens and broilers decreased from 1955–1964 [4]. However, in Japan, the number of layers and broilers raised drastically increased in the 1970s and the 1980s, respectively [4]. Furthermore, the consumption of poultry meat continued to increase, and that of poultry eggs increased in the 1990s and was maintained [5].

Accordingly, the poultry industry is a major agricultural production sector providing food, which is currently the main source of protein in Japan. The objective of this review is to illustrate the recent situation of the Japanese poultry industry, including 1) an overview; 2) situation of the influence of high-pathogenicity avian influenza (HPAI); 3) situation of ingredients for poultry diet; 4) utilization of rice in poultry diet (the reason and research); 5) heat stress in poultry (introduction of research to understand its recent climate effects).

## RECENT OVERVIEW OF THE JAPANESE POULTRY INDUSTRY

**Figure 1:**
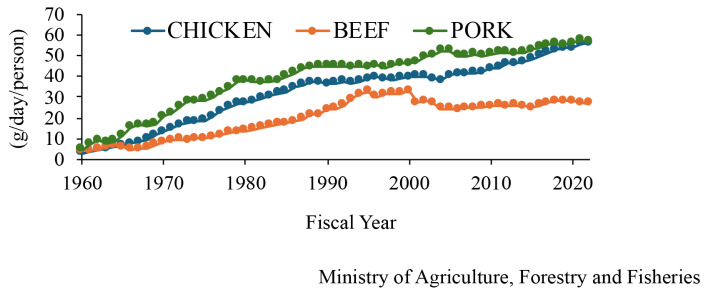
Age-based changes of the consumption of meats of chicken, beef, and pork in Japan (g/d/person).

**Figure 2:**
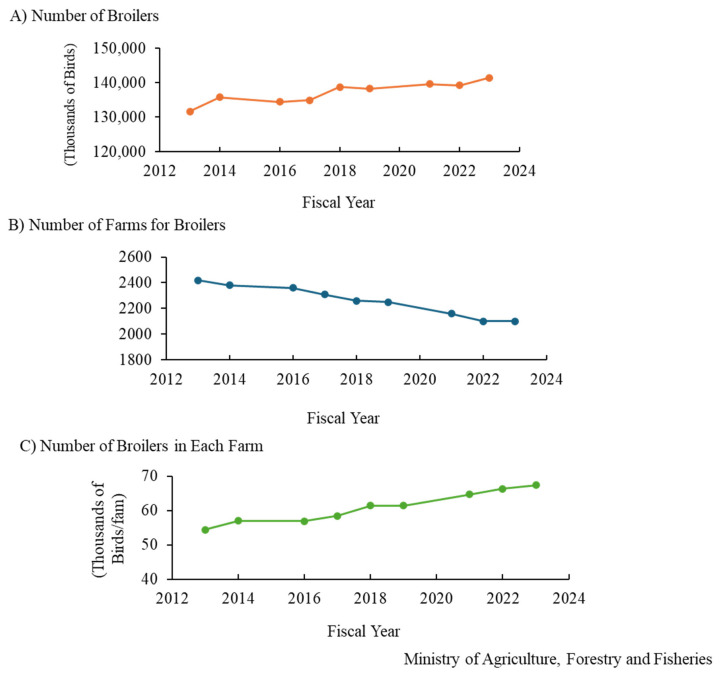
Age-based changes in the number of broilers fed (A), broilers farms (B), and broilers fed in each farm (C) in Japan.

**Figure 3:**
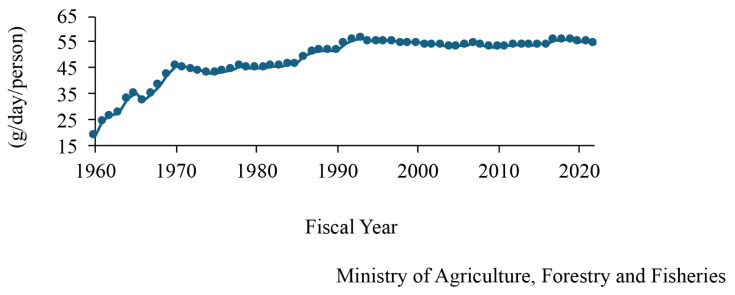
Age-based changes of the consumption of meats of chicken eggs in Japan (g/d/person).

**Figure 4:**
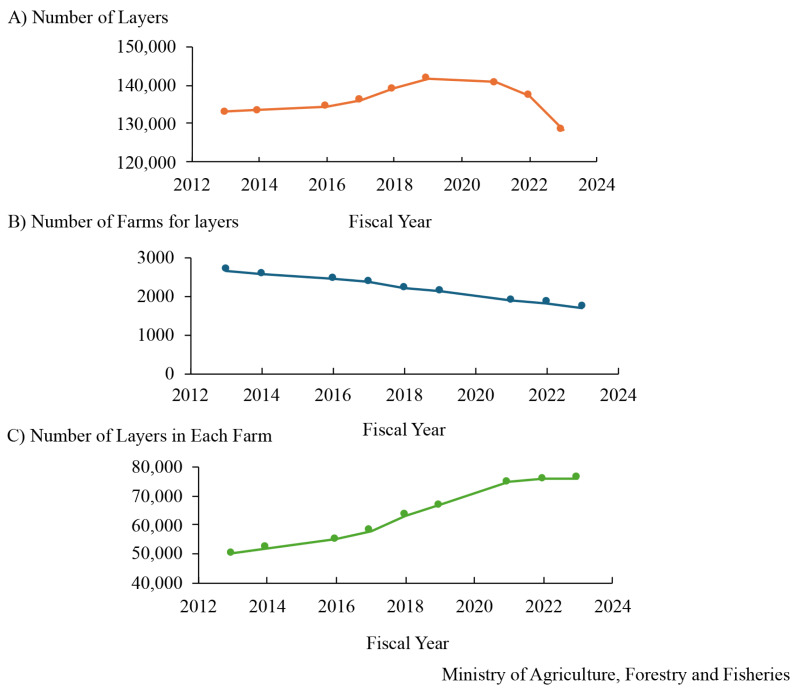
Age-based changes of the number of layers fed (A), layer farms (B), and layers fed in each farm (C) in Japan.

Chicken meat and eggs are popular foods, and meat-type (broiler) and egg-type chickens are raised in Japan, similar to those in other countries. Data on changes in the consumption of chicken meat and eggs, the number of birds fed, and the number of farmers are available on the website of the Ministry of Agriculture, Forestry and Fisheries in Japan [6]. The consumption of broiler meat increased by 12 times in the past 60 years and the per-person consumption of chicken was approximately 60 g/d in the fiscal year 2022 and this increased 12-fold over the past 60 years (Figure 1). In comparison with those of other meats, the consumption of chicken is almost the same as that of pork and twice that of beef. The number of broilers fed in Japan increased slightly to ~140 million in fiscal year 2023 (Figure 2A). Broilers were fed the most in the Kagoshima Prefecture, followed by Miyazaki; these prefectures are located in southwest Japan. However, the number of broiler farms decreased (Figure 2B), and the number of broilers on each farm has recently increased (Figure 2C). Chicken eggs are also consumed in Japan, with the per-person consumption being 55 g/d, and it can be inferred that people consume one egg every day. The consumption of eggs in Japan increased drastically and gradually from fiscal years 1960–1970 and 1978–1993, respectively (Figure 3). The number of layers fed in Japan increased and decreased from fiscal years 2016–2019 and 2021–2023, respectively (Figure 4A). In fiscal year 2022, HPAI erupted in many farms with layers in Japan. Thereby it is assumed that the number of those decreased. The prefecture where the layers were fed the most was Ibaraki, followed by Chiba in fiscal year 2023; these prefectures are located in the central areas of Japan. The number of layer farms also decreased (Figure 4B), similar to that of broiler farms, and the number of layers on each farm recently increased (Figure 4C). Thus, the number of broiler and layer farmers decreased, although the number of birds fed increased, except under the influence of HPAI infection. It is suspected that the cause of this is aging and the lack of poultry farming successors; therefore, this remains a challenge for the Japanese poultry industry, and the development of smart farming technology will be required to deal with this situation.

Poultry products such as meat and eggs were produced in Japan as described above and were also imported. Approximately 560,000 t of chicken meat was imported in fiscal year 2023, and this has changed little over the last 5 years [7]. Chicken meat is also imported and accounts for approximately 25% of chicken consumption in Japan; it is mainly imported from Brazil, Thailand, and the USA. Chicken eggs, both liquid and powder, were also imported from the USA, the Netherlands, and Italy [7]. Recently, approximately 112,000 t of eggs have been imported, which is approximately 4% of the consumption in Japan (based on the conversion of the weight of whole eggs).

## RECENT INFLUENCE OF HPAI IN JAPAN

Similar to that in other countries, the prevalence of HPAI belonging to H5N1 infection affects the price and distribution of poultry products in Japan. The number of HPAI infections in poultry in fiscal year 2022 was 84, which was the worst year; thereafter, it decreased to 11 in fiscal year 2023 [8]. Therefore, the then price of chicken eggs increased to about 1.46 times as the prevalence of HPAI infection increased in many layers. Accordingly, their supplies decreased, and food service industries had to decrease utilization of eggs. Standards of Rearing Hygiene Management for poultry are laid down to prevent infectious diseases in poultry, such as HPAI, by the Ministry of Agriculture, Forestry, and Fisheries based on the Act on the Prevention of Infectious Diseases in Livestock, which was revised and enacted in September 2021. Poultry farmers must manage their farms based on these standards, and accordingly, the basics of livestock health, such as duties of poultry owners and preparation of a rearing hygiene management manual, should be followed to prevent the invasion of pathogens, ensure sanitation, and prevent the dissipation of pathogens from hygienic control areas. However, the prevention of HPAI is challenging, and it remains a hurdle for the poultry industry in Japan, similar to that in other countries. Furthermore, it was found that H5N1 transmit cow-to-cow in USA [9,10]. It was already known that H5N1 could infect mammalian species from birds [10], although the transmission between them was not often found previously [10]. However, H5NI outbreaks in fur farms of Europe and in marine mammals of South America occurred from 2022–2023 [10], and thereafter, those on American daily farms happened in 2024 [9,10]. Though, no reports exist of mammalians H5NI outbreaks until 2024 in Japan, it must be guarded against those incidents. Thus, HPAI outbreaks is globally one of the serious issues currently and a fundamental solution is expected.

## INGREDIENTS OF POULTRY DIET IN JAPAN

Many ingredients of poultry diets are imported to Japan. The distribution of ingredients used for poultry and livestock is presented on the website of the Ministry of Agriculture, Forestry, and Fisheries of Japan [11]. Corn is the most commonly used ingredient in Japanese poultry diets. In fiscal year 2023, approximately 1.8 million (47% of diet) and 3 million tons (51%) of corn were used for broilers and egg-type chickens, respectively. Almost all corn for poultry and livestock diets is imported from foreign countries, such as the USA and Brazil. Therefore, its distribution price is affected by various financial situations globally, such as an increase in the demand for bioethanol from corn and natural disasters. The price of corn increased, subsequently increasing the price of poultry diets from 2020–2021, and it can be assumed that one of the causes for this is the confrontation between Russia and Ukraine. Soybean meal is the second most utilized ingredient in the poultry diet in Japan. In fiscal year 2023, approximately 9.5 (25% of diet) and 8.7 hundred thousand tons (15%) were used for broilers and egg-type chickens, respectively. Almost all soybean meal is imported as soybean or soybean meal from foreign countries, such as the USA, Brazil, and Canada. Therefore, its price distribution is affected by various social factors globally. Other ingredients utilized for poultry diets, such as dried distiller grains with solubles (DDGS) and rapeseed meal, are also imported; therefore, the recent feed self-sufficiency in concentrated feed is less than 15%, challenging the poultry and livestock industry in Japan. Rice is also used as an ingredient for poultry, and approximately 4.1 hundred thousand tons of rice is used for broilers and egg-type chickens each, representing approximately 11% and 7% of their diet, respectively. Rice is widely used as a domestic ingredient in Japan. Research on its use as an ingredient in poultry and livestock diets was conducted in 2010 to promote its use in Japan [12].

### Utilization of rice in poultry diet in Japan

#### Food self-sufficiency in Japan

Based on data from the Japanese Ministry of Agriculture, Forestry and Fishers, Japanese food self-sufficiency decreased from 1965–2022 [13]. Food self-sufficiency based on output in the fiscal year 1965 was 86%, which decreased to 58% in fiscal year 2022, and food self-sufficiency based on calories in fiscal year 1965 was 73%, which decreased to 38% in fiscal year 2022. The reason for this could be the changes in eating habits in Japan. The Japanese have lived on rice, although rice consumption has decreased since fiscal year 1960. It was 350 g/d/person and less than 200 g/person in fiscal years 1960 and 2020, respectively. As it has become possible to choose foods with various grains, such as bread, pasta, or soba, in addition to rice, the use of rice decreased. As the food self-sufficiency of rice is close to 100% in Japan, a reduction in its consumption profoundly affects whole-food self-sufficiency. Based on this reduction, the Japanese government started a policy of reducing acreage under cultivation for rice in fiscal year 1965, to reduce surplus rice and avoid price increases, which increased the number of unutilized paddy fields.

In addition to the reduction in rice consumption, another reason for the lower Japanese food self-sufficiency is an increase in meat consumption, such as chicken. The lifestyle of Japanese people was changing to a Western style, and the consumption of chicken increased approximately 12-fold from fiscal year 1960–2020, based on Japanese data from the Ministry of Agriculture, Forestry and Fisheries. Broilers were fed in Japan, although the parent stock was imported. Almost all the main feed ingredients for broilers, such as corn, soybean meal, and rapeseed meal, were imported. Therefore, an increase in chicken consumption led to a reduction in food self-sufficiency in Japan. Utilization of rice in the diets of poultry and livestock animals is one way to resolve this issue.

### Nutritional value of rice for poultry

The nutritional value of rice is shown in the standard tables of feed composition in Japan (2009) [14]. Crude protein (CP) in dehulled rice and its metabolic energy (ME) in poultry are 7.5% and 3.28 Mcal/kg, respectively. CP and ME in corn is 7.6% and 3.28 Mcal/kg, respectively, implying that the nutritional values of corn and dehulled rice are similar. However, CP and ME in paddy rice is 6.5% and 2.66 Mcal/kg, respectively, i.e., less than those in dehulled rice and corn. Dehulled rice is prepared from paddy rice by thrashing. Therefore, the cost of utilizing paddy rice for the diet is less than that of dehulled rice. Furthermore, it is beneficial to use paddy rice for the diet of broilers. The amino acid composition and availability of rice are also shown in standard tables of feed composition in Japan [14]. Among the essential amino acids for poultry, leucine in dehulled and paddy rice was less than that in corn, and histidine, threonine, and phenylalanine in paddy rice were less than those in dehulled rice and corn. The availability of amino acids in rice and corn did not differ significantly. Therefore, although the amount of amino acids in the diet should be adjusted, the nutritional value in dehulled and paddy rice was sufficient for use in poultry diets.

### Research on broilers fed a rice-based diet

The effects of feeding dehulled or paddy rice to broilers were reported. Dehulled rice can be completely replaced with corn, as shown in the control diet which did not affect the growth of broilers for 7 [15] or 4 weeks [16], or increased their growth performances in starter period [17]. Additionally, the weights of tissues such as the pectoralis superficialis muscle, thighs, heart, liver, abdominal fat, preventriculus, and gizzard did not differ between birds fed a corn main diet and those fed the dehulled rice diet [16]. Therefore, it is possible that dehulled rice can replace corn as an ingredient for broilers.

However, the replacement of corn with paddy rice in starter and grower diets in broilers decreases the growth rate [15]. Body weight and feed intake in broilers fed the paddy rice diet, which replaced all corn in the control diet for 28 d from 11 days-old, were significantly lower than those in the control group, although the feed conversion ratio did not change significantly [16]. Tissue weights of the pectoralis superficialis muscle, thighs, heart, liver, abdominal fat, and proventriculus in broilers fed a paddy rice diet were also significantly lower than those in the control group fed a main corn diet [16]. As poultry comprise gizzards, paddy rice may be digested. Therefore, it is thought that the reduction in growth rate is mainly caused by a reduction in feed intake [16]. When using paddy rice as a replacement for corn in the broiler diet, improved methods, such as crushing and decreasing oil in the diet, may need to be used. Feed intake in broilers fed a crushed paddy rice diet, which was replaced with corn in the control diet, was significantly higher than that in broilers fed untreated paddy rice [16]. Additionally, the weights of the pectoralis superficialis muscle and thighs were slightly higher than those of the untreated paddy rice group [16]. However, as the growth rate was still significantly lower than that in the control group fed the main corn diet, and the process of crushing also increased the cost, this may not be economical. The ME of paddy rice was lower than that of corn and dehulled rice [14]. Therefore, all replacement diets of corn with paddy rice for broilers need to include high levels of oil, implying that the composition of fat in the paddy rice diet is higher than that in the main corn diet. A decrease in the composition of oil in the paddy rice diet was suggested to improve the growth rate of broilers. Feeding of the paddy rice diet, which did not include corn and oil, was the same as that of the main corn diet (control) for 28 d. Broilers grew at almost the same rate as those on the main corn diet. Here, the ME in the paddy rice diet was lower than that in the main corn diet, although the intake of ME did not differ between broilers fed the two diets [16]. Additionally, the paddy rice main diet including same amount of oil as that in the control did not affect body weight of broilers at 42 days-old, although ME and CP were lower than those in the control diet [18]. Source of oil in the paddy rice main diet also affect growth rate in broilers. Broilers fed the paddy rice main diet including high amount (10%) of rendering oil grew as same as those fed the dehulled rice main diet including 5% soybean oils [19]. However, feeding of the paddy rice main diet including 10% soybean or corn oil decreased growth rate in comparison with that of the control fed the dehulled rice main diet including 5% soybean oil. Thus, paddy rice can be used as a feed ingredient in broilers, although the feeding method or composition of the diet should be considered.

Feeding broilers with rice affects meat quality, such as meat color [16,20] and taste [12]. Corn contains high levels of carotenoids such as lutein and zeaxanthin; thus, it contains a yellow-orange pigment. Therefore, feeding broiler chickens with rice is thought to change meat color in comparison with feeding the main corn diet. Feeding dehulled and paddy rice to broilers for 28 d reduced the b* value (yellowness) of the thigh compared with those fed a corn main diet [16]. Additionally, feeding with dehulled rice significantly reduced the b* value in the breast muscle, contrary to feeding with paddy rice. It was also reported that feeding dehulled rice for 21d in the grower period reduced the b* and a* values (redness) and increased the L* value (lightness) in the thigh, although not in the breast [20]. Therefore, feeding broilers with rice may change meat color more in the thighs than that in the breasts.

It is well known that amino acids imparting taste exist and are the taste-active components of meat [21]. Isoleucine, valine, lysine, and arginine impart bitter tastes to beef [22]. Feeding dehulled and paddy rice to broilers, aged 21 d, for 3 weeks increased the composition of free isoleucine, valine, lysine, and arginine in the breast muscle [15]. Additionally, the amount of succinic acid, which has an umami taste [23], in chicken meat ripened for 96 h from broilers fed dehulled rice was significantly higher than that of broilers fed a corn main diet [15]. Therefore, we suggest that feeding rice changes the taste of chicken meat.

### Research on layers fed a rice-based diet

In laying hens, half the replacement of corn by dehulled rice or paddy rice in the diet did not affect the laying rate, average egg weight, feed requirement, or eggshell strength after feeding for 44 weeks [15]. Additionally, half and total replacements of corn by paddy rice in the diet did not affect the laying rate, feed intake, average egg weight, feed requirement, or eggshell strength after feeding for 56 d [15]. Other reports also showed that the replacement of corn with paddy rice did not affect egg production, egg mass, feed intake, feed efficiency, eggshell strength, or eggshell thickness after 10 weeks in laying hens [24]. Similar to that in meat, egg yolk color is also affected by rice-feeding. The yolk color chart values decreased with an increase in the proportion of dehulled rice in the diet [15]. The replacement of corn with paddy rice in the diet from 50% to 100% for laying hens also reduced the value of the egg yolk fan [24]. Thus, dehulled and paddy rice can be used as feed ingredients for laying hens.

The effects of feeding on the intestinal barrier function of rice in poultry were also reported. Murai et al. showed that the replacement of corn in the diet with paddy rice for 14 d in chicks increased intestinal mucin secretion and production by enhancing MUC2 gene expression and epithelial turnover [25]. Additionally, it was shown that feeding paddy rice protects against mucosal disruption more than that by feeding corn by measuring intestinal permeability in dextran sodium sulfate-treated birds [25]. Therefore, it is suggested that the replacement of corn with paddy rice improved intestinal barrier function in poultry.

### Recent situation of rice utilization as a poultry diet and other challenges of Japanese poultry farming in food- and feed self-sufficiency

**Figure 5:**
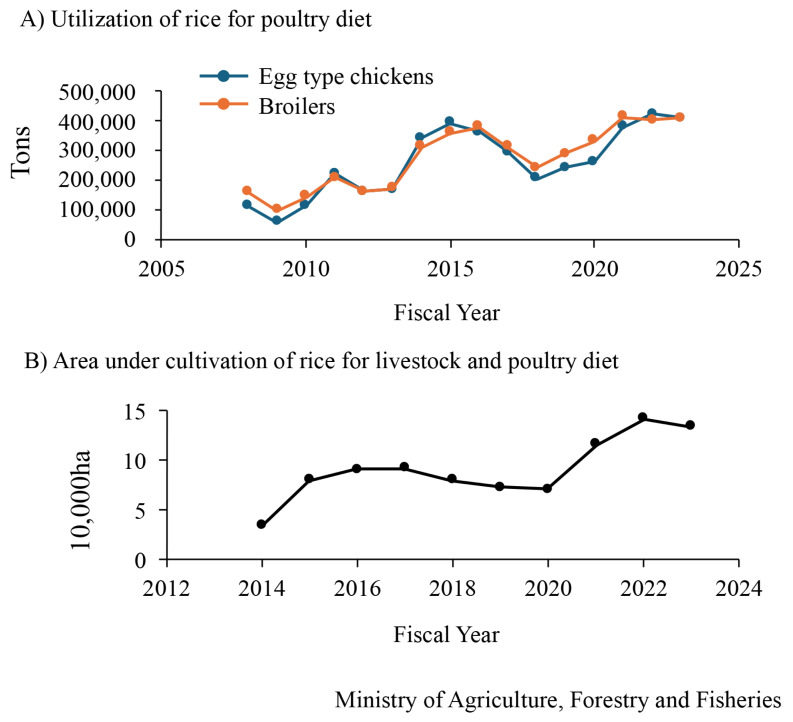
Age-based changes of the utilization of rice for poultry diet (A) and the area under cultivation of rice for livestock and poultry diet (B) in Japan.

The utilization of rice for poultry diet (Figure 5A) and the area under cultivation of rice for livestock and poultry (Figure 5B) increased by approximately 4-fold over the last 10 years. However, food self-sufficiency in Japan remains low. Therefore, methods to improve poultry production should be investigated. Poultry ingredients, such as soybean meal, rapeseed lees, DDGS, and calcium phosphate, are also imported. Thus, the replacement of these ingredients with domestic ingredients should be investigated.

## HEAT STRESS IN POULTRY

### Effect of heat stress on the biological defense system of broilers and its inhibition by rice-feeding

Summer temperatures in Japan were comparatively high, and a recent warning of global warming has been issued. Therefore, the effects of heat stress and the inhibition of its negative effects were investigated. In broilers, a future decline in meat production in Japan was predicted [24]. Analysis using mesh climatic data from the Ministry of the Environment, Government of Japan, and a regression equation, which was calculated from the experiment, showed a possible decrease in meat production, especially in southwest Japan, by 2040, and will be enhanced by 2060 [26]. Almost half of broilers are reared in Kyusyu region, which is in southwest Japan [6], therefore, the future global warming will negatively impact the production of chicken meats in Japan. In fact, high temperature in recent summer has negatively affected broiler faming, such as heat-related death. Farmers insist that protection by the equipment such as cooling pad system may not be enough to inhibit the negative effects of high temperature in summer. As recent mesh climatic data have been upgraded, and it is beneficial to reanalyze them for a more correct prediction of the degree of future decline.

Heat stress modulates the biological defense systems and growth performance of poultry and livestock. It induces oxidative stress in poultry [27]. Excess reactive oxygen species (ROS) such as superoxide, hydrogen peroxide, and hydroxyl radicals are generated by stimuli such as viruses and viral infections [28], imbalance in diet [29], and high temperature, and cannot be detoxified by antioxidative enzymes and antioxidant, leading to oxidative stress in poultry. Several studies reported the induction of oxidative stress at high temperatures in broilers [30–32]. Furthermore, negative effects of heat stress on the immune system were reported [33–36]. Additionally, organs of the immune system, such as the spleen, are atrophied by heat stress in broiler chickens [37,38]. Heat stress induces atrophy of the spleen and modifies cytokine gene expression in broilers. The increase in gene expression of interleukin (IL)- 4 and IL-12 and the decrease of interferon (IFN)-γ mRNA expression in the atrophied spleen by high temperature in broilers were reported [39]. IL-4 is a Th2-type cytokine and acts in humoral immunity. IL-12 is a Th1 type cytokine and IL-12 functions to induce IFN-γ [40], and expression of IFN-γ was decreased under high temperature [39]. Therefore, cytokine signaling may be damaged under high-temperature conditions. Furthermore, we showed that atrophy of the spleen and the increase in gene expression in IL-12 and IFN-γ is not induced by the reduction of energy intake [39]. Modulation of the immune system by heat stress may be inhibited by feeding of rice to broiler chickens [41]. As mentioned earlier, rice can be used as an ingredient in broiler diets. Rice contains tocotrienol and oryzanol [42], which are more common in rice than that in corn [41]. Tocotrienol is a member of the vitamin E family and has high antioxidative activity [43]; it is also known as super vitamin E. Oryzanol also has antioxidative [44] and anti-stress activities [45]. Here, the gene expression of IL-12 in the spleen also increased because of heat stress, and feeding dehulled rice partially inhibited this increase in broilers. Toll-like receptor (TLR) 4, involved in natural immunity, is a ligand of lipopolysaccharide and gram-negative bacteria, and its gene expression is increased by heat stress [41]. The feeding of dehulled rice inhibited its increase at high temperatures. Additionally, plasma immunoglobulin (Ig) M and IgG were increased by heat stress, and feeding dehulled rice partially inhibited the increase in IgM, suggesting an inhibition in the immune response [41]. These increases may be an unnecessary induction of the immune response by high temperature, and feeding dehulled rice may prevent it because the growth rate was enhanced under high temperature in this study [41]. Though the precise mechanism in prevention of heat stress by rice is unknown, a possible speculation exists. As mentioned above, heat stress induces oxidative stress in broilers, and ROS modulates immune system [46] leading to unnecessary induction of immune response such as increase of TLR4 and IgM. Thereafter, energy is utilized for the prevention of modulations, such as increasing of antioxidant capacity. Thereby, energy for growth is reduced by decreasing feed intakes, and growth performance is decreased under heat condition in broilers. As rice includes tocotrienol and oryzanol, oxidative stress and subsequent unnecessary immune response under heat condition are assuaged by those and energy can be used for growth in broilers fed rice. Further research is necessary to prove this speculation. The effects of feeding paddy rice on oxidative stress in broilers under high temperatures were also reported [47]. In acute heat stress, feeding paddy rice inhibited oxidative stress because the increase in malondialdehyde in skeletal muscle induced by high temperature was inhibited by feeding. Additionally, the reduction in the ratio of villus height to crypt depth caused by heat stress was inhibited by feeding paddy rice [47]. However, feeding paddy rice did not influence oxidative stress or intestinal morphology under chronic heat stress. Other feed additives or ingredients also inhibits the reduction of growth performances, immune function, and the induction of oxidative stress. Vitamin E and selenium [48], olive oil, its derivatives [49], and carotenoids such as lycopene [50] could inhibit the negative effects of heat stress in broilers. Thus, nutritional approach to prevent heat stress in broilers were reported. However, inhibiting the negative effects of heat stress completely by nutrition is difficult, as feed intakes are reduced under high temperature conditions. The improvements of housing system or equipment are limited by economic costs. Therefore, genetic selection by tolerance to high temperature in meat-type chickens may be needed to resolve this problem, although the desired outcome would take time. Ultimately, the approach of combining nutrition, housing system, equipment, and breeding improvement with respect to global warming will be required. Currently, almost all broilers in Japan are being imported; however, the production of domestic meat-type chickens tolerant to high temperature by genetic selection may be needed.

### Effect of heat stress in layers

It is thought that high summer temperatures in Japan negatively affect egg production because its reduction is induced by heat stress in laying hens [34]. Layers are reared most in Kanto region in central Japan. However, temperature in summer is high enough to have negative effects on egg production in this region, and laying hens are also reared in southwest Japan. Therefore, an analysis of the degree of future decline in Japan because of global warming, understanding of heat stress in laying hens, and investigation of the prevention of its negative effects are required. High temperatures reduce feed intake and egg production in laying hens, and the immune system is modulated by heat stress [34]. High temperatures increased the egg-breaking rate, indicating that eggshell thickness and strength are reduced by high temperatures. The mechanism includes the respiration rate in laying hens, which is increased at high temperatures to adjust the body temperature, leading to panting, which is a type of hyperventilation. Under panting conditions, the pH of the blood becomes alkaline, indicating induction of alkalosis. [51]. During alkalosis, blood calcium ions decrease, resulting in thin eggshells [52]. These phenomena are known to be the negative effects of high temperatures on laying hens.

A new aspect of the negative effects of heat stress in laying hens was identified, i.e., heat stress induces renal fibrosis in laying hens. Laying hens were fed under thermoneutral or heat stress conditions for 4 weeks [53]. The increment of plasma creatine levels, the decrease of plasma albumin levels, the increments of gene expression of collagen type I alpha, alpha-smooth muscle actin, and transforming growth factor beta in kidney were induced by heat stress in laying hens. The kidneys play an important role in cancer metabolism. Therefore, renal fibrosis may be related to an increase in the number of broken eggs because of heat stress. This effect of heat stress is a novel aspect, and renal fibrosis may be a new target for inhibiting heat stress in laying hens, and the nutritional research related to its inhibition would be needed. However, it is difficult to inhibit negative effects of heat stress completely by nutrition, and the improvements of housing system or equipment are limited by economic costs same as that for broilers. Therefore, genetic selection by tolerance to high temperature in egg-type chickens may be needed, and the approach of combining nutrition, equipment, and breeding improvement with respect to global warming will be ultimately required.

Summarily, the poultry industry has recently gained importance in Japan with increase in product consumption. However, the number of poultry farmers decreased, which is a current challenge. HPAI outbreaks affected the number of chickens fed and the price of poultry products in Japan. Several ingredients of the diet are imported; therefore, their distribution prices are affected by various financial situations globally. This situation negatively affects food self-sufficiency in Japan. Rice is used as a poultry diet in Japan, and its utilization can positively affect food self-sufficiency. However, this is insufficient to increase food self-sufficiency in Japan, and concerns remain regarding the requirements for government subsidies. High temperatures negatively affect poultry meat and egg production in Japan; therefore, the effects of heat stress on broilers and layers and the search for inhibition were investigated. However, no method yet exists to prevent heat stress effects, and further investigations are needed to determine a better method.
